# Institution-Level and Individual Factors Associated with Student Mental Health in Germany: A Multilevel Analysis of StudiBiFra Data

**DOI:** 10.3390/ijerph23070832

**Published:** 2026-06-24

**Authors:** Christiane Stock, Ulrike Grittner, Jennifer Lehnchen, Zita Deptolla, Julia Burian, Katherina Heinrichs

**Affiliations:** 1 Institute of Health and Nursing Science, Charité—University Medical Center Berlin, Corporate Member of Freie Universität Berlin and Humboldt-Universität zu Berlin, 13353 Berlin, Germany; jennifer.lehnchen@charite.de (J.L.); katherina.heinrichs@charite.de (K.H.); 2 Unit for Health Promotion Research, University of Southern Denmark, Degnevej 14, 6705 Esbjerg, Denmark; 3 Institute of Biometry and Medical Statistics, Charité—University Medical Center Berlin, Corporate Member of Freie Universität Berlin and Humboldt-Universität zu Berlin, 13347 Berlin, Germany; ulrike.grittner@charite.de; 4 Health Management, Bielefeld University, 33615 Bielefeld, Germany; zita.deptolla@uni-bielefeld.de (Z.D.); julia.burian@uni-bielefeld.de (J.B.)

**Keywords:** mental health, university students, institutional factors, multilevel analysis

## Abstract

**Highlights:**

**Public health relevance—How does this work relate to a public health issue?**

- Mental health problems are increasing among university students and the pandemic accelerated this trend.
- The institutional context is associated with students’ health and well-being.

**Public health significance—Why is this work of significance to public health?**

- This study addresses a gap in evidence on factors associated with students’ mental health and well-being by focusing on structural factors of higher education institutions, while controlling for relevant individual factors with multilevel models.
- Findings reinforce that gender, age, and study subject play an important role, but also identified that institutional characteristics are associated with students’ mental health and well-being.

**Public health implications—What are the key implications or messages for practitioners, policy makers, and/or researchers in public health?**

- Effective student health promotion should be integrated into continuous organisational processes and address both institutional conditions (e.g., study structures, university culture) and marginalised student groups through low-threshold, discrimination-sensitive services.
- Higher satisfaction with health promotion programmes is associated with lower levels of depressiveness, highlighting the need to improve the quality, accessibility, and student-centred design of these services.

**Abstract:**

While individual determinants of students’ well-being are well established, less is known about the association with the institutional context. This study evaluates institutional-level factors associated with students’ mental health while controlling for individual characteristics. The cross-sectional analysis used data from 12 German institutions (n = 13,715) collected in the StudiBiFra survey on study conditions and student mental health. Individual-level variables included gender, age, study subject group, and four mental health variables (general well-being, depressiveness, cognitive stress, and exhaustion). Institution-level variables comprised institution type, excellence status, multi-campus structure, size, and satisfaction with the quality of health promotion services. Multilevel binary logistic regression models were applied to examine associations between institutional characteristics and mental health outcomes, adjusting for individual factors. Students enrolled at universities of applied sciences showed a lower likelihood of reporting depressiveness and exhaustion. Higher levels of depressiveness and cognitive stress were observed among students at medium-sized institutions compared to small ones. Students not enrolled at institutions with excellence status had lower risks of depressiveness, stress, and exhaustion. Additionally, higher satisfaction with institutional health promotion services was associated with reduced odds of depressiveness. Institutional factors are related to students’ mental health beyond individual characteristics, highlighting the need for a holistic, setting-based approach.

## 1. Introduction

The mental health of students in higher education is a growing concern in many countries [1,2]. In Germany, for instance, a recent meta-analysis showed that one out of five students (21.1%) reports depressive symptoms and that the pooled prevalence rate was higher during the COVID-19 pandemic than before (30.6% versus 18.0%) [3].

Individual-level factors contributing to mental health problems are well understood. Female gender is strongly associated with impaired mental health, both internationally [1] and in Germany [3,4,5]. Students who identify as gender-diverse are at an even higher risk [4,6]. However, this association depends on the mental health problem in focus: female students are more likely to screen positive for internalising disorders like anxiety or affective disorders, whereas male students show more substance use and attention-deficit/hyperactivity disorders [6]. Results on age as a contributing factor to mental health among higher education students remain inconclusive. Auerbach et al. [1] found that older age was a risk-factor for poor mental health in a 12-month period, whereas Mason et al. [6] reported older age to be a protective factor among first-year students in a 12-month period. Students in their first year and those studying medicine or dentistry showed a lower prevalence of depressiveness than those beyond the first year or studying other subjects [3]. Students enrolled in languages/cultural studies, law, and humanities/pedagogy felt the most exhausted, as opposed to students who studied economics, engineering, or arts [5].

However, the association of institutional factors with student mental health is less well understood and little is known about potential variations across the diversity of higher education institutions especially in Germany. The available evidence is predominantly based on studies from the US. Some scholars found that relationships between institutional characteristics and health outcomes including health behaviours existed, but were complex with few clear patterns [7]. Lipson et al. [8] provided the most comprehensive examination of institutional characteristics, finding that doctoral-granting, public, large enrolment, non-residential, less competitive institutions with lower graduation rates were associated with worse mental health outcomes [8]. This suggests that certain institutional structures may increase mental health risk.

The size of the institution also seems to play an important role: a large enrolment was associated with worse mental health with respect to depression and non-suicidal self-injury [8]. In addition, in institutions with higher competitiveness as measured by restricted admission a lower depression prevalence among students was found [8]. The same study also revealed that the institution’s type was related to students’ mental health: relative to students at doctorate-granting institutions, those at master’s colleges and universities were less likely to report non-suicidal self-injury or any mental health problem, and students at private institutions showed lower rates of mental health problems, including suicidal ideation, than those at public ones [8]. While this study provided insights into relevant institutional characteristics, it may not reflect the current situation of student mental health after the pandemic. Another study using the national survey data from the American College Health Association, a survey voluntarily administered by universities and colleges each academic semester to investigate students’ health habits and perceptions, examined the influence of both individual-level and institutional-level characteristics on college students’ stress, psychological distress, and psychological well-being, before and during the COVID-19 pandemic [9]. This study found that studying under COVID-19-related restrictions significantly impacted students’ mental health, but also that institutional-level factors, such as school size or city/town locale, were significant predictors of mental health outcomes [9]. It also demonstrated that school region and religious affiliation were significant predictors of mental health among students [9]. Students enrolled in institutions located in the midwestern, southern, or western regions had lower risks than those attending colleges in the northeastern regions of the country [9]. Additionally, students attending institutions with religious affiliations had higher psychological well-being compared to those that are not religiously affiliated [9].

To conclude, the existing research from the US suggests the need to consider various institutional contexts in efforts to understand predictors of mental health problems and resilience. However, the present evidence on the role of institutional factors for student mental health mainly stems from public and private universities in the US and may not be transferable to other countries and/or the European higher education context, where private universities or those with religious affiliation play a less relevant role in the higher education landscape.

Knowledge on institutional-level factors and contexts is important in order to design health-promoting interventions that take these predictors adequately into account and to adjust evidence-informed programmes and practices to the specific institutional contexts. There is also increasing attention to the importance of preventive and holistic strategies for creating health-promoting environments, such as enhancing the built environment, to better support students’ health and well-being and promote recovery from the pandemic and other stressful events. Both internationally guiding documents, the Okanagan Charter for Health-Promoting Universities and Colleges [10] and the Limerick Framework for Action [11] acknowledge critical environmental determinants, including organisational, social, and physical factors. This calls for a better understanding of the role of institutional and organisational characteristics in promoting student mental health and development.

Therefore, this study aims to examine the associations of institution-level factors (institution type, excellence university, multi-campus institution, institution size, and quality of health promotion services) with student mental health variables, while controlling for individual-level factors in multilevel models. Due to the sparse knowledge in the field, our hypotheses are limited to assuming that students enrolled at smaller institutions and at those with higher institution-level satisfaction with health promotion services may report better mental health.

## 2. Materials and Methods

### 2.1. Data Collection

Data were collected as part of the survey on study conditions and mental health among university students (acronym StudiBiFra), a nationwide collaborative project conducted by Charité—University Medical Center Berlin and Bielefeld University. Cross-sectional online surveys were carried out between June 2021 and March 2023 at 13 German public higher education institutions. For this analysis, we included student data from 12 institutions (10 universities and 2 universities of applied sciences). Due to a missing variable at one of the institutions, we included only data from students who reported their gender identity as an important control variable in the analysis resulting in a sample of 13,715 students. Throughout the manuscript, we use the term ‘university’ for both types unless we focus on differences between the two types of higher education institutions.

Students at each institution were invited to participate through multiple channels, including email, institution websites, flyers, and social media, with recruitment coordinated locally by each institution. The survey was administered using LimeSurvey (LimeSurvey GmbH, Hamburg, Germany). Before beginning the survey, all participants provided informed consent and were able to complete the questionnaire in either German or English, except at one institution where the survey was offered only in German. The overall response rate was 11%.

The study was conducted in accordance with the principles of the Declaration of Helsinki. Ethical approval was granted by the Ethics Committee of Charité—University Medical Center Berlin (26 March 2021; No. EA1/055/21).

### 2.2. Questionnaire

The Bielefeld Questionnaire on Study Conditions and Mental Health was applied in this study. The questionnaire is an established instrument that is available for universities to assess student satisfaction with their study conditions and to monitor the mental and general health, as well as well-being, of students [12,13]. In order to ease the interpretation of the data derived and to provide university leadership and student health management programmes with data to prioritise health-promoting intervention and programme targets, the entire questionnaire uses a 5-point Likert-type answering format ranging from 1 to 5. For the reporting of the survey results, positive statements are assigned higher numerical values, whereas negative statements receive lower numerical values. Positive values can be interpreted as resources. In contrast, low values indicate burdens or strains. Overall, mean values for single items or scales are classified into three categories: burdens (mean between 1.00 and 2.50), values with potential for development (mean between 2.51 and 3.49), and resources (mean between 3.50 and 5.00) according to Burian et al. [12]. The questionnaire was validated and pretested at one faculty of Bielefeld University with 264 participants.

#### 2.2.1. Sociodemographic Variables

Students were asked to report their gender identity by selecting one of three response options (“female,” “male,” or “gender-diverse”) or by choosing “no answer” and their age. Age was categorised into <26, 26–30, 31–40, and >40 years of age.

Each participating institution provided its own list of study programmes and/or faculties. For the purpose of data aggregation, these programmes were grouped into five categories: education, humanities, social, behavioural and media sciences, economics, law, languages, arts, and culture (“humanities”); natural and life sciences, mathematics, and statistics (“natural sciences”); engineering, technology, and architecture (“engineering”); medicine and health sciences (“health”), and “other”. For details of the study programmes summarised under each category, see Appendix A.

#### 2.2.2. Mental Health Variables

Four scales assessed mental health: general well-being (3 items), depressiveness (5 items), cognitive stress (4 items), and exhaustion (3 items). All items were introduced with the question: “How often did the following statements apply to you during the last two months of your studies?” Responses were recorded on a 5-point Likert-type scale ranging from “(almost) always” to “(almost) never.”

General well-being: One of the three items in this scale was specifically developed for this study, while the other two were taken from the Productivity and Social Capital in Business (ProSoB) questionnaire [14]. The Cronbach’s α was 0.80 in the current sample.

Depressiveness: The five items measuring depressiveness were derived from the ProSoB questionnaire [14]. The scale demonstrated high reliability (Cronbach’s α = 0.90 in the current sample).

Cognitive stress: The four items assessing cognitive stress were adopted from the Copenhagen Psychosocial Questionnaire (COPSOQ) [15], showing a Cronbach’s α of 0.86 in the current sample.

Exhaustion: The three items used to measure exhaustion were adapted from the ProSoB questionnaire [14], with acceptable reliability in the current sample (Cronbach’s α = 0.76).

For each mental health dimension, mean scale scores were calculated. If fewer than one-third of the items within a scale were missing, missing values were replaced by the mean of the remaining items in that scale. If more than one-third of the items were missing, no scale mean was calculated. Applying the questionnaire cut-offs described above [12], the scale means of the depressiveness, cognitive stress, and exhaustion scales were then categorised into high level (mean between 1.00 and 2.50 = 2) versus medium to low levels (mean between 2.51 and 5.00 = 1). In addition, the mean for the general well-being scale was categorised into high level (mean between 3.50 and 5.00 = 2) versus medium to low levels (mean between 1.00 and 3.49 = 1).

#### 2.2.3. Institution-Level Factors

In addition to data collected from students, metadata were collected from representatives of each institution. Based on these metadata, we included the following institution-level variables in the analysis. These data included the type of institution (university or university of applied sciences), the geography of the campus regarding the number of campus sites (single-campus or multi-campus institution), whether the institution was awarded an excellence seal by the German excellence initiative at the time of the data collection, and the number of enrolled students in bachelor and master programmes. Based on student number, and similar to Suárez-Reyes et al. [16], the institutions were categorised into small (<10,000), medium (10,000–20,000), and large institutions (>20,000).

In order to include a variable that captures the satisfaction with the quality of health promotion services at the institution level, we created a mean score of the student responses to the question: “How satisfied are you with the health promotion offers of your university (e.g., university sports programme)?” The response options ranged from 1 (“very dissatisfied”) to 5 (“very satisfied”). Mean values of individual student responses were calculated for each institution.

### 2.3. Statistical Analysis

Depending on the type of variable, descriptive data are reported as absolute and relative frequencies or as means with standard deviations. In addition, we fitted separate multilevel binary logistic regression models to examine the associations between individual student and university characteristics and four mental health outcomes. All models included the same set of fixed-effect independent variables (gender, age, field of study, institution type, excellence university (yes/no), multi-campus university (yes/no), institution size, and health service offerings) and accounted for clustering at the institutional level through random intercepts for universities. The binary outcome variables represented high levels of overall well-being (Model 1), depressiveness (Model 2), cognitive stress (Model 3), and exhaustion (Model 4). All models were estimated using binary logistic mixed models.

Because the between-university variance components were very small and sometimes not estimable in the frequentist mixed-effects models, we relied on Bayesian estimation instead for Model 1, Model 3, and Model 4. In these three models, we used Bayesian models, where we specified weakly informative priors (Normal (0, 2.5) for fixed effects and Exponential (1) for random effect standard deviations) and ran four chains for 2000 iterations each (1000 warmup) to ensure convergence and reliable posterior distributions. We report estimates and 95% confidence or credible intervals (CI) depending on the framework used. All analyses were conducted in R Version 4.5.2 [17]. Bayesian models were estimated using the brms package Version 2.23.0. [18].

## 3. Results

### 3.1. Characteristics of the Sample

As shown in Table 1, the sample is characterised by a majority of students being female, under 26 years of age, and studying a subject in the “humanities” group.

The percentage of students with a high level of mental health problems ranged from 34.6% of students with a high level of cognitive stress, 35.6% with a high level of depressiveness, to 43.6% with a high level of exhaustion. Two-thirds of students (66.7%) had medium to low overall well-being.

The vast majority of students was studying at a university (90.9%), at an institution with multiple campus sites (93.7%), and at an institution with more than 10,000 students (85.4%). More than one-third (36.2%) were enrolled at an institution with excellence seal.

### 3.2. Individual and Institution-Level Variables Associated with Depressiveness

Table 2 presents the results of a multilevel logistic regression analysis examining individual and institution-level factors associated with depressiveness among students (n = 13,715).

At the individual level, gender identity was associated with depressiveness. Compared with female students, male students showed lower odds of reporting depressiveness (OR = 0.85; 95% CI: 0.78–0.92). In contrast, students identifying as gender-diverse had substantially higher odds of depressiveness (OR = 2.63; 95% CI: 2.00–3.46). Age differences were also observed. Students aged 26–30 years had slightly higher odds of depressiveness compared with those younger than 26 years (OR = 1.18; 95% CI: 1.07–1.29), whereas students older than 40 years had markedly lower odds (OR = 0.52; 95% CI: 0.39–0.70). No substantial differences were found for students aged 31–40 years compared to the youngest age group. With regard to subject groups, most fields did not differ substantially from students in the “humanities” group. However, students in health-related disciplines had lower odds of depressiveness (OR = 0.55; 95% CI: 0.47–0.64).

At the institutional level, several structural characteristics of universities were associated with depressiveness. Students enrolled at universities of applied sciences had lower odds of depressiveness than those at universities (OR = 0.75; 95% CI: 0.60–0.94). Similarly, studying at an institution not classified as an excellence university was associated with lower odds of depressiveness (OR = 0.53; 95% CI: 0.44–0.63). The variable indicating whether an institution operated across multiple campuses showed somewhat lower odds of depressiveness, but this result was not statistically robust. Institution size was also associated with depressiveness: students at medium-sized universities (10,000–20,000 students) had almost twice the odds of depressiveness compared with those at small universities (OR = 1.98; 95% CI: 1.62–2.42), while students at large universities (>20,000 students) also showed higher odds (OR = 1.30; 95% CI: 1.06–1.60). Finally, higher institutional-level satisfaction with health promotion services was associated with lower odds of depressiveness (OR = 0.73; 95% CI: 0.54–0.99).

### 3.3. Individual and Institution-Level Variables Associated with Overall Well-Being, Cognitive Stress, and Exhaustion

Table 3 presents the results of the Bayesian multilevel models examining associations between sociodemographic and institution-level characteristics and three mental health outcomes: well-being, cognitive stress, and exhaustion.

At the individual level, several sociodemographic characteristics were related to these mental health variables. Compared with female students, male students reported higher levels of well-being (OR = 1.60; 95% CI: 1.47–1.74) as well as lower levels of cognitive stress (OR = 0.60; 95% CI: 0.55–0.65) and exhaustion (OR = 0.63; 95% CI: 0.59–0.69). In contrast, students identifying as gender-diverse reported lower well-being (OR = 0.31; 95% CI: 0.21–0.47) and higher levels of cognitive stress (OR = 2.24; 95% CI: 1.71–2.91) and exhaustion (OR = 1.82; 95% CI: 1.39–2.38) compared with female students.

Differences were also observed across age groups. Students aged 26–30 years reported lower well-being (OR = 0.74; 95% CI: 0.67–0.82) and higher levels of cognitive stress (OR = 1.24; 95% CI: 1.12–1.36) and exhaustion (OR = 1.39; 95% CI: 1.26–1.52) compared with students younger than 26 years. Among students aged 31–40 years, well-being was also lower than in the youngest age group (OR = 0.78; 95% CI: 0.68–0.90), while cognitive stress was similar and exhaustion was higher (OR = 1.37; 95% CI: 1.20–1.56). In contrast, students older than 40 years reported higher well-being (OR = 1.38; 95% CI: 1.07–1.76) and lower levels of cognitive stress (OR = 0.51; 95% CI: 0.38–0.67) than the youngest age group, whereas exhaustion did not differ substantially.

Variation across subject groups was also apparent. Compared with students in the “humanities” group, those studying engineering reported higher levels of well-being (OR = 1.23; 95% CI: 1.04–1.47). Students in health-related disciplines additionally showed higher levels of well-being (OR = 1.46; 95% CI: 1.26–1.69) and lower levels of cognitive stress (OR = 0.60; 95% CI: 0.52–0.70) and exhaustion (OR = 0.71; 95% CI: 0.62–0.83), while students in natural sciences reported slightly higher exhaustion (OR = 1.12; 95% CI: 1.02–1.24). For the remaining subject groups, differences across the outcomes were small.

Regarding institution-level characteristics, most variables showed limited associations with the mental health outcomes. Students studying at universities of applied sciences reported lower exhaustion than those studying at universities (OR = 0.62; 95% CI: 0.43–0.90), whereas the associations with well-being and cognitive stress were less pronounced. Not attending an excellence university was associated with lower levels of cognitive stress (OR = 0.67; 95% CI: 0.53–0.84) and exhaustion (OR = 0.70; 95% CI: 0.52–0.97) compared to attending a university with excellence status, while the relationship with well-being was less clear. No substantial differences were observed between multi-campus and single-campus universities for the three mental health outcomes. Some variation was observed with regard to institution size. Students enrolled at medium-sized universities reported somewhat higher cognitive stress compared with those at small universities (OR = 1.31; 95% CI: 1.00–1.68), whereas differences in well-being and exhaustion were less consistent. Finally, institution-level satisfaction with health promotion services showed no clear associations with well-being, cognitive stress, or exhaustion.

Overall, the estimated between-institution variance components were small, indicating that only a minor proportion of the variance in these mental health outcomes was attributable to differences between universities.

## 4. Discussion

This study was aimed at examining institution-level factors (institution type, excellence university, multi-campus institution, institution size, and satisfaction with the quality of health promotion services) as factors associated with student mental health variables, while controlling for relevant individual-level factors. The results demonstrate that the individual-level factors gender, age, and study subject have a strong and consistent impact on indicators of student mental health, while institution-level factors play a less prominent role.

In this analysis, we did not attempt to include all individual-level factors potentially associated with student mental health; rather, we focused on the factors gender, age, and study subject group, which were earlier identified as highly important predictors [3,4,5]. This selection was made in order to adjust the multilevel analysis for those sociodemographic factors that most profoundly shape the composition of the student body of higher education institutions.

### 4.1. Individual-Level Factors and Mental Health

Regarding the influence of gender identity, the results of our models show that even when controlled for institutional-level factors, male students reported consistently better mental health than their female peers. This was reflected in a higher likelihood for high well-being and a lower likelihood for depressiveness, stress symptoms, and exhaustion. The findings support earlier studies showing that female students report poorer mental health than male students both in Germany [5,19] and internationally [6]. This nonetheless overlooks the fact that male students often score higher in substance use and hyperactivity disorders [6], which were variables not in the focus of our study. Another finding in our analysis was that students that identify as gender-diverse had consistently poorer mental health than those identifying as male. This difference was profound, with up to three times higher odds for the risk of depressiveness in gender-diverse students as compared with female and male students. Our findings are in line with other research showing that transgender and gender-diverse young people often experience high rates of mental health problems [20].

Age was another important and consistent predictor of student mental health. We identified a non-linear relationship between age and mental health indicators. Compared with the youngest age group (<26 years), students of middle age (26–30 years) were less likely to show good mental health with respect to all outcomes. For the older students (31–40 years), the associations were mixed, and for the students above 40 years of age the associations showed the opposite direction: students of a relatively older age had a higher likelihood of experiencing good mental health for all indicators, except exhaustion. This finding is worrisome, as it shows that when students grow older their mental health tends to deteriorate unless they belong to the small group (2.3% in our sample) that are aged 40 or above. This is in line with a longitudinal study showing a worsening of students’ mental health during the first years of studies among medical students [21], which might be explained by increasing family care tasks, but also by the accumulation of academic and institutional stressors occurring when students progress in their studies.

Further, the study subject is also associated with students’ mental health. While studying natural sciences and technology or engineering did not make a consistent difference when compared to the students studying any of the subject in the humanities, social science, and arts group, students studying health-related subjects stood out: consistently, across all mental health indicators, they were less likely to report poor mental health. Better health of students in health subjects as compared to students in other subjects is also reported by others [3]. This finding may be explained by a higher level of health literacy and preventive knowledge among students studying health subjects, because these are topics taught during their studies. However, a selection towards students with higher level of resilience may also contribute to the difference observed. The majority of students studying a health-related subject in Germany are medical students, because most of the other health professions educations (such as nursing and physiotherapy) are rarely taught at universities or universities of applied sciences, but mostly at professional schools. Entering a medical programme in Germany, however, usually requires excellent average grades at high-school level and the selection of students in medical programmes is very high towards high performing resourceful students. Thus, students with mental health difficulties may be less likely to fulfil the strict entry requirements. This means that while gender and age can be regarded as predictors of students’ mental health, the causal path does not seem to be clear regards study subject. Studying a health-related subject may influence the mental health of students positively, but any pre-existing mental health condition may also influence whether young people enter a health-related subject.

### 4.2. Institution-Level Factors and Mental Health

While most of the findings discussed above are consistent with previous research on predictors of students’ mental health, our study reveals fundamentally new insights into the role of institutional factors on students’ mental health in Germany. Most of the institutional-level factors that we could include into our analysis showed at least some associations with mental health indicators. Even though none of the institution-level factors (institution type, multi-campus institution, excellence university, size in terms of student number, and satisfaction with the quality of health promotion services) showed a consistent association with all mental health indicators, three of the factors showed associations with more than one of the outcomes, namely, type of institution, size, and excellence status.

With respect to institution type, students nested in universities of applied sciences had lower likelihoods for depressiveness and exhaustion than students at universities. To our knowledge, no other studies have explored differences in students’ mental health between these two types of higher education institution. Explanations for our findings may include that the programmes at universities of applied sciences are typically more structured, with fixed schedules, smaller classes, and closer contact with lecturers. These institutions also emphasise practical training, internships, and clear career pathways, which can reduce uncertainty about future employment. This interpretation is consistent with previous findings from the StudiBiFra Study showing higher satisfaction with study conditions among students at universities of applied sciences compared to those enrolled at universities [13]. Together, this may lower stress and improve students’ overall well-being. It is also conceivable that students with impaired mental health might choose a university of applied sciences above a university in order to benefit from the specific study conditions in this type of universities.

The other factor with an impact on more than one mental health outcome was the size of the institution. In line with our hypothesis, the results showed higher levels of depressiveness and cognitive stress for students enrolled at medium-size compared with small institutions (<10,000 students). For large institutions (>20,000 students), such increased risk was only found for depressiveness, though. Earlier research showed similar results with poorer mental health for students in high enrolment institutions [8,9]. Similar to universities of applied sciences, smaller institutions may be able to provide better guidance and support for students and prevent alienation and feelings of anonymity, with positive effects on stress and depression. Therefore, smaller universities might be especially attractive to students with mental health risks.

As a third factor, students not enrolled in institutions with excellence status awarded in the German excellence initiative showed lower risks for depressiveness, stress, and exhaustion. This is somewhat in contrast with results from Lipson et al. [8] showing that in institutions with higher competitiveness, a lower depression prevalence among students was found. Explanations for our findings may include that the institutions with excellence status put a high emphasis on research excellence, but are less focused on quality of teaching and may neglect attention to a health-conducive environment for students. However, self-selection effects may also play a role for this finding.

Even though one may assume that a campus geography with more than one campus site could cause stress and may diminish social well-being, the analysis did not show an association of campus type with any of the mental health indicators. However, one more institution-level factor showed an association with mental health, though with depressiveness only. This factor was student satisfaction with the offer of health promotion services as an institutional mean. Since the mean differed slightly between institutions, the analysis showed that at institutions with a higher mean satisfaction with health promotion offers, the odds for depressiveness among students were lower. This result is in line with our hypothesis and provides an important new insight, because promoting students’ health with a campus-wide health promotion programme is not well evaluated and data showing any improvement in students health when programmes are implemented are sparse [16,22]. Even though isolated well-being interventions show promising effects in improving students’ mental health (e.g., [23]), the overall investments of universities in health-promoting programmes and offers for students lack evidence of effectiveness. Our result at least indicates that when student satisfaction with the health promotion offer is higher across institutions, this is reflected in lower level of depressiveness in the respective student population. This finding may encourage universities to enhance and improve their health promotion programmes.

### 4.3. Limitations

Even though we regard the multi-site data collection resulting in a large and diverse dataset and the collection of institution-level data as a strength of the study, several limitations need to be considered. First, the data were based on a convenience sample, and selection bias cannot be excluded due to the relatively low response rates typical of student surveys. Although participating institutions implemented tailored recruitment strategies to maximise participation, the resulting sample may be unbalanced and therefore is not representative of the overall university student population in Germany. Female students are overrepresented in the dataset, with 67% in contrast to only 51% in the general German student population (https://de.statista.com/ accessed 16 June 2026). On the other hand, the dataset is fairly representative regarding the age distribution, with 70% of students under 26 years in our sample and 62% in the general student population (https://de.statista.com/ accessed 16 June 2026). Second, the cross-sectional design precludes causal inference even though several of our independent variable are given (e.g., age) and an impact of mental health on them is less likely. Third, as the data relied on self-report, misreporting of mental health indicators due to social desirability bias cannot be ruled out.

Although the study assessed four mental health variables—an advantage compared with studies focusing on a single indicator—the validated scales used do not provide clinical cut-offs to estimate the prevalence of specific conditions. Missing scale items were handled using person-mean imputation to minimise case loss; while alternative imputation approaches might have yielded slightly different estimates, any resulting deviations are likely to be small given the large sample size.

Our analyses would have certainly benefitted from a higher number of institutions in the dataset. Since only data from 12 institutions could be included, the variation of these institution-level variables in the sample is limited, which is indicated by the very small between-university variance. Therefore, a larger variation of institutions may have resulted in more conclusive findings (e.g., with respect to multi-campus environments) on their association with individual-level mental health outcomes. In addition, more structure-level factors could be analysed with a dataset comprising a higher number of institutions, such as geographical location or location in different federal states.

### 4.4. Future Research Directions

We could only include a limited number of institution-level characteristics that we could retrieve from the participating study sites, which limits the conclusions that can be drawn from this study. Therefore, future research should expand the multilevel analyses to include more institution-level factors such as geography, campus ecology, and architecture, and more detailed aspects of implemented health policies and practices, and should, at the same time, assess their impact not only on mental health but also on physical, social, and behavioural aspects of students’ health. Furthermore, institutional factors associated with symptoms of anxiety as one of the major mental health problems among students should also be studied in future research. Beyond the broad institution-level structural factors examined in our analysis, it would also be relevant to study their interplay with other factors shaping the study environment of students such as social relationships, academic pressures, or institutional culture that are also known to be associated with students’ mental health [13].

We recommend regular monitoring of students’ health in Germany at all higher education institutions in order to identify students’ health needs and potential effects of implemented measures that would also enable the identification of institution-level factors with much higher statistical power.

### 4.5. Future Directions for Health-Promoting Campuses

Our findings suggest that measures to promote student health at universities should, to maximise effectiveness, address two levels. First, particular attention should be paid to marginalised student groups by providing low-threshold, discrimination-sensitive support services. Second, interventions should target institutional framework conditions, which shape broader aspects of university culture and therefore operate at the level of structural prevention. Such structural measures require a long-term perspective, as changes to study conditions typically involve complex organisational processes.

However, our analyses also identified factors influencing students’ mental health that cannot be modified through organisational change, such as institutional size and type. The results therefore support a stronger emphasis on promoting students’ mental health and well-being in medium- and large-enrolment institutions, and in universities rather than universities of applied sciences, both at the policy level—for example, within federal state ministries of education—and within the institutions themselves.

A key finding of our study for the practice of health-promoting universities is that higher levels of institutional satisfaction with health promotion programmes were associated with lower levels of depressiveness. Although causal inferences cannot be drawn due to the cross-sectional design, this result nevertheless underscores the importance of systematically improving the quality, visibility, and alignment of such services with students’ needs. It also supports the assumption that effective communication about these services may itself be beneficial: students feel recognised, taken seriously, and supported by their university. This, in turn, can foster a sense of belonging and reduce feelings of isolation. The adage “Do good and communicate it” may therefore serve as a useful guiding principle for those engaged in student health promotion.

## 5. Conclusions

This study found that students enrolled at universities of applied sciences and at institutions without excellence status showed an overall lower risk of mental health problems. In contrast, students at medium-sized institutions reported higher levels of depressiveness and cognitive stress as compared to students at small ones, while greater satisfaction with institutional health promotion services was associated with a lower likelihood of depressiveness. Overall, the finding that institution-level factors are associated with students’ mental health over and above individual-level factors reinforces the holistic settings approach, which constitutes a central principle of the Okanagan Charter [9]. Within this framework, universities are explicitly conceptualised as ‘settings’ for health and well-being. Investing in health-conducive environments is even more important for universities that are large or have a strong focus on research excellence.

In light of an increasing number of global crises, health promotion in higher education can play a crucial role in reducing students’ worries and uncertainties, strengthening their overall well-being, and fostering awareness of mental health as a key issue among future leaders who may act as multipliers in their professional contexts.

## Figures and Tables

**Table 1:** Characteristics of the sample (n = 13,715).

Variable		n	% ^1^ or Mean (SD)
Individual-level variables			
Gender identity			
Female		9193	67.0
Male		4272	31.1
Diverse		250	1.8
Age group			
<26		9502	69.7
26–30		2593	19.0
31–40		1217	8.9
>40		318	2.3
Subject group			
Humanities		7834	59.3
Natural sciences		2481	18.8
Engineering		1289	9.8
Health		1379	10.4
Other		229	1.7
Overall well-being			
Low to medium		8978	66.7
High		4479	33.3
Depressiveness			
Low to medium		8602	64.4
High		4747	35.6
Cognitive stress			
Low to medium		8795	65.4
High		4644	34.6
Exhaustion			
Low to medium		7553	56.4
High		5833	43.6
Institution-level variables			
Institution type	Number of institutions		
University	10	12,469	90.9
University of applied sciences	2	1246	9.1
Excellence university			
No	9	8754	63.8
Yes	3	4961	36.2
Multi-campus institution			
No	2	868	6.3
Yes	10	12,847	93.7
Institution size			
Small (<10,000 students)	5	1996	14.6
Medium (10,000–20,000 students)	4	3719	27.1
Large (>20,000 students)	3	8000	58.3
Institution-level satisfaction with health promotion services	12	13,715	3.78 (0.24)

^1^ Valid percentages reported; sums might not add to 100% due to rounding error.

**Table 2:** Results of multilevel binary logistic regression analysis on individual and institution-level variables associated with depressiveness (n = 12,795 individuals at 12 universities), frequentist approach.

Variable	OR	95% CI
Individual-level variables		
Gender identity		
Female	1.00	—
Male	0.85	0.78, 0.92
Diverse	2.63	2.00, 3.46
Age group		
<26	1.00	—
26–30	1.18	1.07, 1.29
31–40	0.94	0.81, 1.07
>40	0.52	0.39, 0.70
Subject group		
Humanities	1.00	—
Natural sciences	0.96	0.87, 1.06
Engineering	0.99	0.84, 1.16
Health	0.55	0.47, 0.64
Other	1.11	0.81, 1.52
Institution-level variables		
Institution type		
University	1.00	—
University of applied sciences	0.75	0.60, 0.94
Excellence university		
Yes	1.00	—
No	0.53	0.44, 0.63
Multi-campus institution		
No	1.00	—
Yes	0.77	0.58, 1.02
Institution size		
Small (<10,000 students)	1.00	—
Medium (10,000–20,000 students)	1.98	1.62, 2.42
Large (>20,000 students)	1.30	1.06, 1.60
Institution-level satisfaction with health promotion services (per point increase)	0.73	0.54, 0.99

Note. OR = Odds ratio; 95% CI = 95% Confidence interval with random intercepts for institution.

**Table 3:** Bayesian multilevel models on individual and institution-level variables associated with mental health variables.

Variable	Well-Being n = 12,898		Cognitive Stress n = 12,877		Exhaustion n = 12,827	
	OR	95% CI	OR	95% CI	OR	95% CI
Individual-level factors						
Gender identity						
Female	1.00	—	1.00	—	1.00	—
Male	1.60	1.47, 1.74	0.60	0.55, 0.65	0.63	0.59, 0.69
Diverse	0.31	0.21, 0.47	2.24	1.71, 2.91	1.82	1.39, 2.38
Age category						
<26	1.00	—	1.00	—	1.00	—
26–30	0.74	0.67, 0.82	1.24	1.12, 1.36	1.39	1.26, 1.52
31–40	0.78	0.68, 0.90	0.94	0.81, 1.08	1.37	1.20, 1.56
>40	1.38	1.07, 1.76	0.51	0.38, 0.67	0.87	0.67, 1.13
Subject group						
Humanities	1.00	—	1.00	1.00	1.00	—
Natural sciences	1.03	0.93, 1.15	1.00	0.90, 1.10	1.12	1.02, 1.24
Engineering	1.23	1.04, 1.47	0.94	0.79, 1.11	0.94	0.80, 1.11
Health	1.46	1.26, 1.69	0.60	0.52, 0.70	0.71	0.62, 0.83
Others	0.96	0.69, 1.32	1.36	0.99, 1.84	0.88	0.65, 1.20
Institution-level factors						
Institution type						
University	1.00	—	1.00	—	1.00	—
University of applied sciences	1.52	0.88, 2.89	0.79	0.61, 1.03	0.62	0.43, 0.90
Excellence university						
No	1.00	—	1.00	—	1.00	—
Yes	1.61	0.94, 2.60	0.67	0.53, 0.84	0.70	0.52, 0.97
Multi-campus institution						
No	1.00	—	1.00	—	1.00	—
Yes	0.80	0.41, 1.45	1.05	0.78, 1.50	1.16	0.79, 1.72
Institution size						
Small (<10,000)	1.00	—	1.00	—	1.00	—
Medium (10,000–20,000)	0.72	0.43, 1.24	1.31	1.00, 1.68	1.40	0.97, 1.90
Large (>20,000)	0.99	0.57, 1.73	1.10	0.86, 1.41	0.97	0.67, 1.37
Institution-level satisfaction with health services (per point increase)	1.29	0.43, 3.67	0.81	0.54, 1.22	1.11	0.60, 2.20
Between-institution variance of the model	0.072, SD: 0.269, ICC: 0.022.		0.006, SD: 0.075, ICC: 0.002.		0.016, SD: 0.128, ICC: 0.005.	

Note. OR = Odds Ratio; 95% CI = 95% Credible interval, random intercept for institution; ICC = Intraclass correlation; SD = standard deviation.

## Data Availability

Data supporting reported results are not publicly accessible due to contracted restrictions by the single higher education institutions that participated in this study. Access can only be provided upon reasonable request.

## Abbreviations

The following abbreviations are used in this manuscript:

StudiBiFra	Survey on study conditions and mental health

## Appendix A. Description of Study Programmes Summarised Under the Study Programme Categories

Label Humanities: Education, Humanities, Social, Behavioural and Media Sciences, Economics, Law, Languages, Arts and Culture ○ Education (Education science; Training for pre-school teachers; Teacher training without subject specialisation; Teacher training with subject specialisation). ○ Arts (Audio-visual techniques and media production; Fashion; Interior and industrial design; Fine arts; Handicrafts; Music and performing arts). ○ Humanities (Religion and theology; History and archaeology; Philosophy and ethics). ○ Languages (Language acquisition; Literature and linguistics). ○ Social and behavioural sciences (Economics; Political sciences and civics; Psychology; Sociology and cultural studies). ○ Journalism, media and communication studies; and information studies (Journalism and reporting; Media and communication studies; Library; Information and archival studies). ○ Business and administration (Accounting and taxation; Finance; Banking and insurance; Management and administration; Marketing and advertising; Secretarial and office work; wholesale and retail sales; work skills) ○ Law
Label Natural Sciences: Natural and life sciences, mathematics and statistics ○ Biology; biochemistry; environmental sciences; Natural environments and wildlife; Chemistry; Earth sciences; Physics; Mathematics and statistics.
Label Engineering: Engineering, Technology and Architecture ○ Information and communication technologies (Computer use; Database and network design and administration; Software and applications development and analysis). ○ Engineering and engineering trades (Chemical engineering and processes; Environmental protection technology; Electricity and energy; Electronics and automation; Mechanics and metal trades; Motor vehicles; ships and aircraft). ○ Manufacturing and processing (Food processing; Materials (glass; paper; plastic and wood); Textiles (clothes; footwear and leather); Mining and extraction). ○ Architecture and construction (Architecture and town planning; Building and civil engineering). ○ Agriculture, forestry, fishery, and veterinary sciences (Crop and livestock production; Horticulture; Forestry; Fisheries; Veterinary).
Label Health: Medicine and health sciences ○ Health (Medicine; Public health and Health sciences; Dental studies; Nursing and midwifery; Medical diagnostic and treatment technology; Therapy and rehabilitation; Pharmacy; Traditional and complementary medicine and therapy). ○ Hygiene and occupational health services (Community sanitation; Occupational health and safety).
Label: Other - Cross-disciplinary studies
